# Learnings from user feedback of a novel digital mental health assessment

**DOI:** 10.3389/fpsyt.2022.1018095

**Published:** 2022-10-20

**Authors:** Erin Lucy Funnell, Benedetta Spadaro, Jiri Benacek, Nayra A. Martin-Key, Tim Metcalfe, Tony Olmert, Giles Barton-Owen, Sabine Bahn

**Affiliations:** ^1^Cambridge Centre for Neuropsychiatric Research, Department of Chemical Engineering and Biotechnology, University of Cambridge, Cambridge, United Kingdom; ^2^Psyomics Ltd., Cambridge, United Kingdom; ^3^Independent Researcher, Cambridge, United Kingdom

**Keywords:** digital health, mental health, mHealth, user feedback, thematic analysis, composite international diagnostic interview

## Abstract

Digital mental health interventions (DMHI) have the potential to address barriers to face-to-face mental healthcare. In particular, digital mental health assessments offer the opportunity to increase access, reduce strain on services, and improve identification. Despite the potential of DMHIs there remains a high drop-out rate. Therefore, investigating user feedback may elucidate how to best design and deliver an engaging digital mental health assessment. The current study aimed to understand 1304 user perspectives of (1) a newly developed digital mental health assessment to determine which features users consider to be positive or negative and (2) the Composite International Diagnostic Interview (CIDI) employed in a previous large-scale pilot study. A thematic analysis method was employed to identify themes in feedback to three question prompts related to: (1) the questions included in the digital assessment, (2) the homepage design and reminders, and (3) the assessment results report. The largest proportion of the positive and negative feedback received regarding the questions included in the assessment (*n* = 706), focused on the quality of the assessment (*n* = 183, 25.92% and *n* = 284, 40.23%, respectively). Feedback for the homepage and reminders (*n* = 671) was overwhelmingly positive, with the largest two themes identified being positive usability (i.e., ease of use; *n* = 500, 74.52%) and functionality (i.e., reminders; *n* = 278, 41.43%). The most frequently identified negative theme in results report feedback (*n* = 794) was related to the report content (*n* = 309, 38.92%), with users stating it was lacking in-depth information. Nevertheless, the most frequent positive theme regarding the results report feedback was related to wellbeing outcomes (*n* = 145, 18.26%), with users stating the results report, albeit brief, encouraged them to seek professional support. Interestingly, despite some negative feedback, most users reported that completing the digital mental health assessment has been worthwhile (*n* = 1,017, 77.99%). Based on these findings, we offer recommendations to address potential barriers to user engagement with a digital mental health assessment. In summary, we recommend undertaking extensive co-design activities during the development of digital assessment tools, flexibility in answering modalities within digital assessment, customizable additional features such as reminders, transparency of diagnostic decision making, and an actionable results report with personalized mental health resources.

## Introduction

Mental health disorders are among the leading causes of disability worldwide, as indicated by disability-adjusted life-years (1) and are associated with substantial detrimental impacts on the individual (2–4), societal, and economic level (5, 6). The prevalence of mental health disorders is estimated to be rising, with a 21% increase of people accessing mental health services in the United Kingdom between 2016 and 2019 (7), with further reported increases in the wake of the COVID-19 pandemic (8–13). The demands on mental healthcare services are high, with a large number of patients presenting to services, alongside a parallel increase in patients with complex or severe psychiatric symptoms (13, 14). Despite the high prevalence and burden of mental health disorders globally, a lack of adequate recognition of disease and delivery of traditional mental health care persists (15–17) due to barriers such as the high costs and an understaffed work force (14, 18).

Considering these barriers to traditional mental healthcare, digital mental health interventions (DMHI) provide a method to support detection, treatment, and management of psychiatric conditions (16, 19, 20). DMHIs are versatile as they can be integrated in the traditional care pathway, and used alongside in-person care (20–22). Thus, DMHIs and traditional in-person care should be viewed as complementary. DMHIs are able to address geographical and service level barriers to accessing mental health services, such as understaffing and medical coverage of more remote areas (23) providing support to healthcare providers. In return, healthcare providers can facilitate engagement with DMHIs, as evidence shows that two-thirds of individuals prescribed a digital intervention for depressive or anxiety symptoms by their GP reported using it (24). Additionally, DMHIs can address non-service level barriers associated with accessing traditional face-to-face mental health care, such as patients experiencing difficulties or distress in disclosing mental health concerns, or perceived stigmatization (23, 25, 26).

In the context of identification and triage of mental illness, digital screening has the potential to reduce the number of patients who require healthcare appointments by identifying patients who may benefit from signposting to self-help strategies or to digital interventions for management and treatment of symptoms, and do not require high-intensity treatment from a clinician (23). These benefits are compounded by a documented interest in the implementation of digital questionnaires designed for the assessment of mental health disorders. These tools are the second most commonly reported digital tool used by General Practitioners (GPs), with computerized cognitive behavioral therapy (cCBT) reportedly being the most commonly used (27). Importantly, an assessment component of mental health apps is perceived positively within written user reviews for such apps (28).

Perhaps most crucially, when considering possible benefits, digital mental health assessments have been shown to improve identification of mental health disorders (24, 25). Previous work has demonstrated that, of those participants who underwent digital screening for common psychiatric disorders in a primary care setting, a quarter of those identified as experiencing depressive or anxiety symptoms were previously unidentified by their GP. The majority of these previously unidentified patients were experiencing mild to moderate symptom severity, however 1-in-10 presented with severe symptoms or reported current suicidal ideation at the time of screening (24). Additionally, digitized screening may help in removing clinician variability or error by standardizing the questions asked of patients when assessing psychiatric symptoms (29, 30). Furthermore, screening ensures that differential mental health conditions are considered during more comprehensive psychiatric assessments. For instance, evidence indicates that most patients with bipolar disorder seek help during depressive rather than manic episodes (31). This can result in a major depressive disorder misdiagnosis, and result in inappropriate or ineffective treatment being delivered to the patient. By ensuring that a history of past mania episodes is detected and considered during clinical decision making, patients may be diagnosed earlier and more accurately, and will thus receive effective treatment earlier. This would be a major benefit as research indicates a high rate of mis- and non-identification of mental health diagnoses, with a meta-analysis including 50,731 patients showing that GPs accurately diagnosed only 47 percent of depression patients (32). Evidence also shows that a GP’s ability to correctly detect depression varies dependent on symptom severity, with greater difficulty in accurately identifying mild to moderate presentations (33). An even greater rate of mis-diagnosis is observed in bipolar disorder of just under 70 percent, with the majority of patients being mis-diagnosed with unipolar depression (34, 35). This misidentification rate contributes to documented delays of up to 10 years in receiving an accurate diagnosis of bipolar disorder (34–37). Delays to accurate diagnosis are associated with significant costs to the individual, such as a poorer response to treatment (38), and increased risk of both substance misuse (39) and suicide attempts (40, 41). Reducing these delays by implementing accurate screening tools for bipolar disorder into the care pathway could minimize individual suffering associated with misdiagnosis and delays to receiving the correct treatment, as well as conferring cost savings to healthcare systems (42). For these reasons, there is a compelling health, societal, and economic case to be made for mental health assessments that can improve diagnostic accuracy and early identification.

Digital mental health assessments are generally perceived positively by their users. However, despite the promise of DMHIs for both care providers and patients there persists low user engagement and high levels of dropout (43, 44). This has prompted research into reasons for poor user engagement. User attitudes toward and perspectives of digital mental health tools have been investigated via interviews and focus groups (45–49), surveys (50–53), and app user reviews studies (54–58). In a systematic review of 208 studies investigating user engagement with DMHIs, three constructs of engagement factors were identified related to: (1) the user, (2) the program, and (3) the technology and environment (59). Relevant for the current paper are engagement factors related to the program construct, and the technology and environment construct. These engagement constructs include specific potential barriers such as the usability, the impact of the DMHI on the user, and the perceived helpfulness of the DMHI (59). Critically, potential user-centric barriers to user engagement include the perceived usability and usefulness of the DMHI. Understanding user perspectives of digitally delivered mental health tools, for example from feedback or during a co-design process, can ensure that the resulting digital tool is suitable for the target user groups’ requirements and preferences (60).

Considering the importance of investigating user perspectives, the current study utilized feedback provided by users from a large-scale pilot study of a novel digital mental health assessment to explore users’ perspectives. The data used for the current study was taken from the Delta Study conducted by the Cambridge Centre for Neuropsychiatric Research (CCNR) between April 2018 and February 2020 (61). Briefly, the Delta Study was designed to: (1) identify patients with bipolar disorder from a group of patients misdiagnosed with major depressive disorder (MDD); and (2) improve the accuracy and speed of MDD diagnosis among low mood individuals. During the Delta Study, participants completed a novel digital mental health assessment, with a sub-set further providing dried blood spot samples in order to identify proteomic biomarkers which may differentiate between MDD and bipolar disorder. Those who provided blood spot samples were additionally invited to complete the Composite International Diagnostic Interview 3.0 (CIDI; 62). The CIDI was developed by the World Health Organization with the view to extend the scope to include diagnostic criteria from the International Classification of Disease in order to facilitate international comparative research (62). The CIDI has been demonstrated to have excellent concordance with the SCID for bipolar spectrum disorders (63), whilst simultaneously offering more flexibility regarding delivery than the SCID, as the CIDI can also be delivered by a trained and supervised layperson (64). Therefore, the CIDI was considered an appropriate tool for the objectives of the Delta Study.

Following the completion of the novel digital mental health assessment, participants were sent a non-diagnostic results report which outlined the most likely conditions the participant may be experiencing, based on their answers. After the receipt of this results report, participants were provided a feedback survey designed to collect information on their motivations to engage with such an assessment, their perception of the worthwhileness of completing the assessment, and actionable constructive feedback which can be operationalized by the developers to improve future iterations of the assessment. This feedback represents a wealth of information that can guide future development of digital psychiatric assessments to best drive user engagement. Whilst the feedback survey focused on collecting the participant perspectives of, and areas of future improvement for the novel digital mental health assessment, as well as the Delta Study in general, some users provided un-prompted feedback about the CIDI. Thus, we analyzed the feedback dataset in order to: (1) identify features of the digital mental health assessment which users perceived as either positive or negative; and (2) identify commonly mentioned features of the CIDI delivered via telephone, from any unprompted CIDI feedback. Based upon our findings related to each of these aims, recommendations were devised to offer developers insight into optimizing the user experience of digital self-assessments for mental health.

## Materials and methods

### Data collection

The data used for the current study were taken from the Delta Study. For a detailed description of the Delta Study methods see Olmert et al. (61).

**FIGURE 1:**
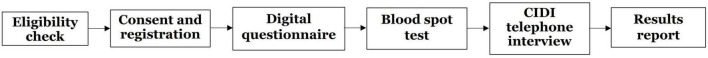
Delta Study flow.

Recruitment was performed via Facebook advertisements and email mailing lists comprised of individuals who had consented to be recontacted in the context of previous CCNR studies. During recruitment, participants were familiarized with information regarding the key objectives of the Delta Study in the landing pages outlining the aims, the organizers and funders, the stages of the which the participant would be invited to complete (see Figure 1 for a breakdown of the flow of the Delta Study), details about what the user can expect to receive following their participation [i.e., a results report with indicated mental health conditions, links to sources of help (SOH) and some brief psychoeducation]. Through a “frequently asked questions” link, the participants were given details about the novel digital assessment including the length of the assessment, the expected time to complete the assessment, and how the mental health symptom data they provide is confidentially stored.

**FIGURE 2:**
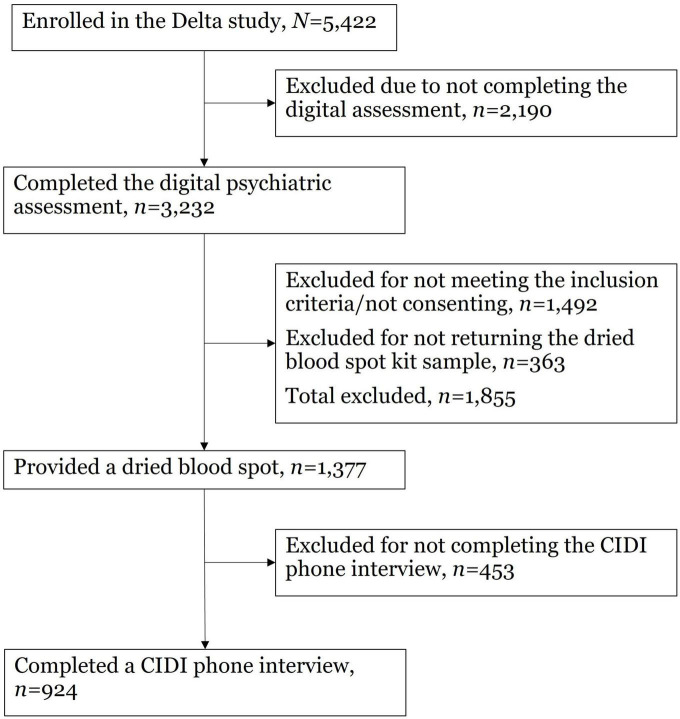
Flowchart of participants through the Delta Study.

Three groups of patients with current depressive symptoms were recruited into the Delta Study: (1) individuals with no lifetime diagnosis of a mood disorder including MDD and bipolar disorder; (2) individuals with a diagnosis of MDD within the past 5 years and no lifetime diagnosis of bipolar disorder; and (3) individuals with a previous bipolar disorder diagnosis. Inclusion criteria for the Delta Study were: (1) aged 18−45; (2) currently living in the United Kingdom; (3) currently experiencing at least mild depressive symptoms as indicated by a PHQ-9 score of at least 5 (65); (4) no current suicidality; and (5) not currently breastfeeding or pregnant. A total of 5,422 participants were enrolled in the Delta Study. The Delta Study was a three-part study: (1) a digital diagnostic assessment; (2) a dried blood spot collection kit, delivered by post; and (3) Composite International Diagnostic Interview (CIDI) (62), delivered via the telephone. Only participants who returned a dried blood spot sample were invited to participate in the CIDI. Of the enrolled sample, 924 participants completed all three parts of the Delta Study. See Figure 2 for a breakdown of participant flow through the Delta Study.

Following the eligibility check, consent and registration, participants accessed the Delta Study webpage hosting the novel digital assessment. The digital assessment was developed as a webpage designed for use on both PC and smartphone. The digital assessment was comprised of six separate question sessions: (1) socio-demographics, validated measures including the Warwick-Edinburgh Mental Wellbeing Scale (WEMWBS; 66), and psychiatric history; (2) current and past manic and hypomanic symptoms; (3) current and past depressive symptoms; (4) personality traits based on the big five personality framework (66); (5) treatment history, alcohol and substance use; and (6) screening for other psychiatric symptoms. Questions included in question sessions 2, 3, and 6 were based on existing validated measures (62, 67–77) and the expertise of a practicing psychiatrist (SB). Input from a panel of ten patients with lived experience of psychiatric disorders was also implemented when developing the digital assessment. This patient input included review of questions, the results report, and the website and flow of participants through the Delta Study. This patient input resulted in amendment to aspects included in the novel digital assessment (i.e., questions and results report), and participant facing materials included in the Delta Study.

The digital assessment used a logic model which only displayed questions to participants which were relevant based on their previous answers. The potential maximum number of questions answered by a participant was 382, and the average number of questions answered by participants was 284. Upon enrollment, participants were invited to create an account in order to be able to take breaks during the assessment, while automatically saving their progress.

After completion of the digital assessment, participants were sent a brief results report. The results report included: (1) screening results; (2) personalized psychoeducation; (3) a list of SOH; and (4) general self-help tips. The results included in the results report were reached by using a novel algorithm based on the diagnostic rules as set out in the Diagnostic and Statistics Manual of Mental Health Disorders, Fifth Edition (DSM-5; 69).

A feedback questionnaire was available immediately after users completed the digital assessment and after they received their results report. Completion of the feedback questionnaire was optional. The feedback questionnaire was comprised of four closed questions and four open questions designed to gather constructive criticism from users in order to improve future iterations of the digital assessment (see Table 1).

**TABLE 1:** Questions included in the Delta Study feedback questionnaire.

(1) How did you hear about the Delta trial?
(1) An advert on Facebook
(2) In a doctor’s surgery
(3) Through my support group
(4) A family member told me
(5) A friend told me

(2) In general, did you find participating in the Delta trial to be worthwhile?
(1) Not at all
(2) To a small extent
(3) Somewhat
(4) Very much so

(3) The questions are essential for our future diagnostic accuracy. Do you have any thoughts about the design, wording or flow of the questions to help us improve them?
*(Free text box)*

(4) We want to make the trial process as smooth and easy for you as possible. What did you think of the homepage design and our email reminders?
*(Free text box)*

(5) Finding out what you liked or disliked in your results report will help us make it more relevant and helpful. Do you have any comments about your results report?
*(Free text box)*

(6) *(only for participants who completed the blood spot kit)* Did you have any issues with the blood spot kit?
(1) No
(2) Yes *(If yes – free text box)*

(7) We’re interested in what motivated you to take part in the Delta trial. Why did you decide to participate? Select all that apply.
(1) To contribute to mental health research
(2) I was curious about the trial
(3) To learn more about my mental health
(4) To learn more about my mental health in general

(8) We’d love to hear anything else you’d like to share:
*(Free text box)*

Participants were eligible to participate in the CIDI interview if they (1) consented to provide a dried blood spot for biomarker analysis; (2) did not suffer from a blood-borne infectious disease; and (3) had no previous diagnosis of schizophrenia. The dried blood spot collection kit was designed to identify protein biomarkers, previously identified as associated with the psychiatric disorders MDD, bipolar disorder, and schizophrenia (78).

The CIDI was conducted via a telephone call by CIDI-certified interviewers who received continued mentoring and training. Only the modules required for a lifetime mood disorder diagnosis were offered. There were six possible outcomes of the CIDI interview: bipolar disorder type I, bipolar disorder type II, sub-threshold bipolar disorder, MDD, MDD with subthreshold bipolar disorder, and no mood disorder.

Participants were sent a digital follow-up and usefulness questionnaire at 6 and 12 months after the initial digital mental health assessment. The purpose of the follow-up questionnaire was to investigate help-seeking behaviors since the Delta Study, as well as any changes in mental health diagnosis or psychiatric treatment, and to evaluate the perceived usefulness of participation in the Delta Study. The two validated measures, the WEMWBS (79) and PHQ-9 (65) were included in the initial digital assessment and in both the 6-month and 12-month follow up in order to analyze wellbeing scores and depressive symptoms longitudinally, and to determine whether engaging with a digital assessment was associated with improvements in either of these outcomes.

### Data selection

The 40.35% of participants who completed the digital assessment (*n* = 1304) also answered the feedback questionnaire. All the participants who completed the feedback questionnaire were included in the dataset.

As the primary aim of the current study was to use thematic analysis to investigate positive and negatively perceived features of a digital mental health assessment, only feedback questions capturing feedback on the question wording, design and flow, homepage design and reminders, and the results report were included (Table 1; questions 3, 4, and 5). The decision was made to exclude general feedback (Table 1; question 8) in order to avoid the inclusion of data not specifically commenting on the digital assessment (i.e., general feedback on the Delta Study, information the participant wished to share about their personal mental health). Only feedback about the digital mental health assessment itself was included in the thematic analysis. Therefore, any general feedback about the Delta Study pilot or the blood spot kit was not included in the analysis. Any feedback text which only conveyed a sentiment (i.e., “was good,” “I liked it”), or any feedback text which was unclear as to what it was referencing (i.e., “it was smooth” which could refer to the trial itself, the question flow or the assessment design) were considered “Not Applicable” for the current study and were excluded from the thematic analysis.

There were 918 responses to the question about assessment design, 1058 responses to the question about homepage design, and 937 responses to the question about the results report design.

Feedback data on the CIDI was included in the current study only when the feedback was specifically commenting on the interview itself and not its delivery (i.e., the skill of the interviewer, the ease or difficulty of booking the interview). Feedback comments were considered to be referring to the CIDI if they mentioned being read the questions rather than digitally delivered, or included the words: (1) “Telephone;” (2) “Interview;” (3) “Telephone question(s);” (4) “Clinical interview.”

### Data analysis

Participant characteristics and psychiatric history were gathered by the novel digital mental health assessment utilized in the Delta Study. Descriptive analyses of this information were conducted to determine averages and frequencies, as appropriate. Group differences between Delta Study participants who did and did not provide feedback were calculated. Mann−Whitney *U*-tests were used to examine group differences in continuous variables because the data were non-normally distributed. Group differences in categorical variables were calculated using chi-square or Fisher exact test.

The thematic analysis was manually conducted in Excel, following the Braun and Clarke framework (80). The feedback comments were read and re-read until the first author (EF) was familiar with them and any initial ideas were noted. Initial codes were created (EF). For feedback about the novel digital assessment, codes were grouped into “positive” and “negative” sentiment groups. For the CIDI, the decision was made not to group codes into sentiment due to the unprompted nature of the feedback which minimizes the amount of data and its representation in the dataset. The codes were added to a coding framework with brief descriptions for each. The feedback comments were then manually allocated codes under blinded conditions (EF/BS/JB) guided by the coding framework. Any inconsistencies in the code allocations between the authors (EF/BS/JB) were discussed until a consensus was reached. In order to reduce review bias and increase the robustness of results, a double independent review approach was utilized. Therefore, all feedback comments included in the thematic analysis received their final coding based on the consensus of at least two independent reviewers.

The identified codes were then grouped into broader themes, independently by two reviewers (EF/BS), which were then discussed with the third reviewer (JB) until consensus was reached.

Once the thematic labeling was finalized and code/theme frequencies had been calculated, the frequency of overlapping themes was calculated in Excel (see Supplementary material 2−6). This involved determining which themes were commonly reported together within the user feedback for each question prompt asked.

## Results

### Demographics

Participants who completed the feedback survey were mostly female (*n* = 939, 72.01%), with one or more previous diagnosis (*n* = 1015, 77.84%). 289 (22.16%) participants who completed the feedback survey reported no previous diagnosis. 722 (55.37%) participants who completed the feedback survey reported two or more previous diagnoses. The most commonly reported previous diagnosis of participants who completed the feedback survey was MDD (Table 2). The mean PHQ-9 score of the participants who completed the feedback survey indicates the sample group experienced moderate MDD symptom severity (Table 2).

**TABLE 2:** Demographic information about patients who completed the Delta Study feedback questionnaire (*n* = 1304).

	Provided feedback in the Delta Study (*n* = 1304)	Did not provide feedback in the Delta Study (*n* = 1928)	*U*	*P*	*r*	Chi-square (*df*)	φ _*c*_
**Age, years**							
Mean (SD)	29.53 (7.66)	27.94 (7.11)	1110024.50	<0.001	0.10	N/A	N/A
**Sex**							
Male, *n* (%)	365 (27.99)	542 (28.11)	N/A	0.940	N/A	0.006 (1)	0.001
Female, *n* (%)	939 (72.01)	1386 (71.89)	N/A		N/A		
**Education^A^**							
<GCSE or equivalent, *n* (%)	41 (3.14)	54 (2.80)	N/A	0.680	N/A	2.304 (4)	0.027
GCSE or equivalent, *n* (%)	222 (17.02)	344 (17.84)					
Advanced level or equivalent, *n* (%)	403 (30.90)	555 (28.79)					
Undergraduate degree, *n* (%)	439 (33.67)	678 (35.17)					
Postgraduate degree, *n* (%)	199 (15.26)	297 (15.40)					
**WEMWBS**							
Mean (SD)	34.66 (7.99)	34.52 (8.08)	1245297.00	0.651	N/A	N/A	N/A
**PHQ-9^B^**							
Mean (SD)	14.64 (5.17)	14.77 (5.17)	1240539.50	0.525	N/A	N/A	N/A
**Previous diagnosis**							
MDD, *n* (%)	959 (73.54)	1275 (66.13)	N/A	<0.001	N/A	20.024 (1)	0.079
Bipolar, *n* (%)	109 (8.36)	121 (6.28)	N/A	0.024	N/A	5.106 (1)	0.040
GAD, *n* (%)	599 (45.94)	826 (42.84)	N/A	0.082	N/A	3.019 (1)	0.031
OCD, *n* (%)	95 (7.29)	131 (6.79)	N/A	0.592	N/A	0.288 (1)	0.009
PD, *n* (%)	136 (10.43)	194 (10.06)	N/A	0.735	N/A	0.114 (1)	0.006
SAD, *n* (%)	264 (20.25)	329 (17.06)	N/A	0.022	N/A	5.254 (1)	0.040
An eating disorder, *n* (%)	110 (8.44)	140 (7.26)	N/A	0.022	N/A	1.503 (1)	0.220
A personality disorder, *n* (%)	140 (10.73)	167 (8.66)	N/A	0.048	N/A	3.894 (1)	0.035
Schizophrenia, *n* (%)	2 (0.15)	8 (0.41)	N/A	0.333	N/A	1.725 (1)	0.023
**Self-rated quality of mental health**							
Poor, *n* (%)	918 (70.40)	1310 (67.95)	N/A	0.139	N/A	2.185 (1)	0.026
Fair, *n* (%)	321 (24.62)	500 (25.93)	N/A	0.399	N/A	0.712 (1)	0.015
Good, *n* (%)	65 (4.98)	118 (6.12)	N/A	0.171	N/A	1.878 (1)	0.024

GCSE, General Certificate of Secondary Education; MDD, major depressive disorder; GAD, generalized anxiety disorder; OCD, obsessive-compulsive disorder; PD, panic disorder; SAD, social anxiety disorder. ***(A)*** GCSE and Advanced level are academic qualifications taken by secondary education students in the United Kingdom, in the 11th and 13th year of education, respectively. ***(B)*** The PHQ-9 score can be used to indicate the level of depression severity. Scores of 0−4, 5−9, 10−14, 15−19, and 20 or above indicate a severity of minimal, mild, moderate, moderately severe, and severe respectively (65).

Analysis of group differences between Delta Study participants who did and did not provide feedback found that the groups did not significantly differ in terms of sex, educational attainment, total WEMWBS score, total PHQ-9 score, or self-rated mental health quality (Table 2). Delta Study participants who provided feedback were significantly older than those who did not provide feedback. There were differences in the psychiatric histories of Delta Study participants who did or did not provide feedback, with a significantly higher proportion of individuals in the group who did not provide feedback reporting a previous diagnosis of MDD, bipolar disorder, social anxiety disorder, an eating disorder, or a personality disorder (Table 2).

**FIGURE 3:**
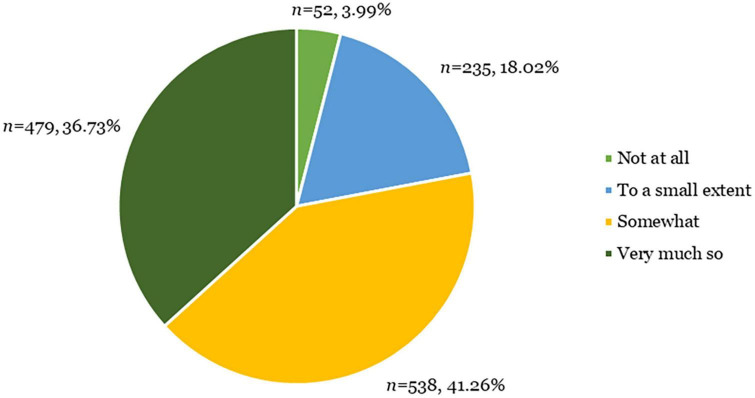
Worthwhileness scores from users who provided feedback on the Delta Study (*n* = 1304).

Participants additionally provided scores on the level of how worthwhile they considered participating in the Delta Study (Table 1; question 2). The current study determined a mean worthwhileness score of 3.11 (*SD* = 0.83) on a 1 to 4 scale, with a score of 3 translating to the user considering participation in the study to have been at least somewhat worthwhile. Most participants (*n* = 1017; 77.99%) considered completing the digital assessment to be at least somewhat worthwhile (Figure 3).

### Thematic analysis

Example feedback comments presented in the results were extracted from the dataset. The example feedback comments included in the results were taken verbatim from the dataset, so any spelling or grammatical errors are as intended.

#### Feedback on the design, wording, or flow of the questions

**FIGURE 4:**
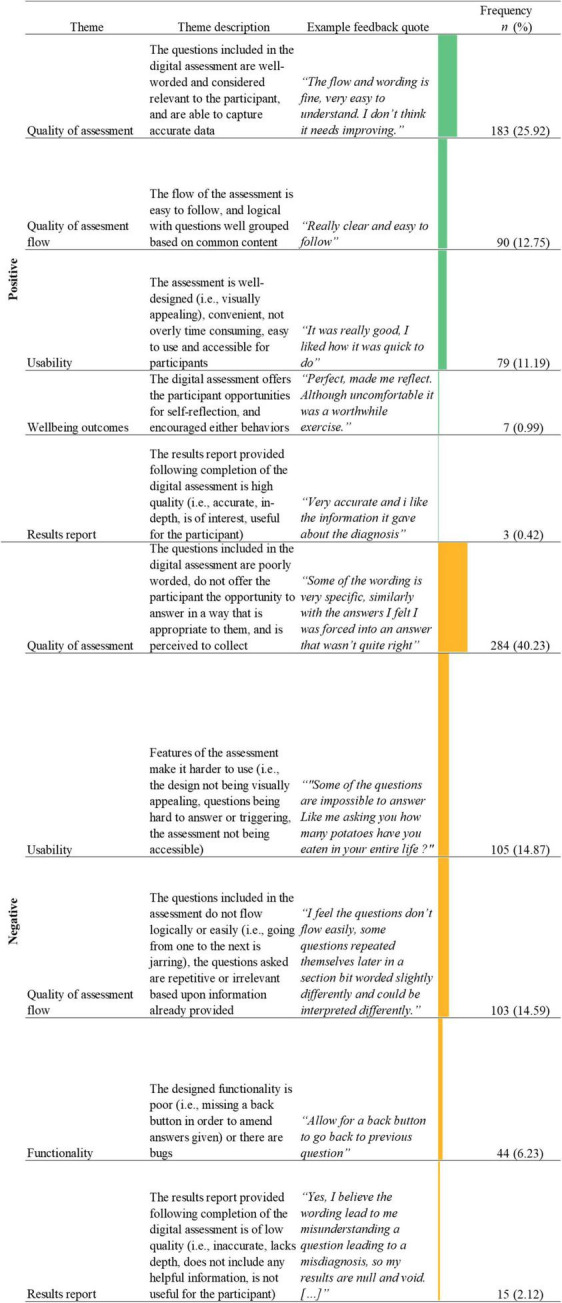
Name, description, examples, and frequencies of themes as identified in the thematic analysis of feedback responses about the question design, wording, and flow (*n* = 706; See Supplementary material 1 for novel digital assessment theme count). Bars represent theme frequency as a percentage of the total number of relevant feedback responses (*n* = 706).

212 (23.09%) responses to the question regarding the design, wording, or flow of the questions were identified as being “Not Applicable” (N/A) to the aims of the current study. This left a total of 706 relevant responses. The average word count of the included feedback for this question was 21.87 (*SD* = 33.89). In total, 10 themes were identified from feedback comments on the design, wording, or flow of questions included in the Delta assessment (See Figure 4), including three negative major themes and three positive major themes, focused specifically upon the quality of the assessment content, the quality of the assessment flow, and the usability.

The quality of assessment, related to the question wording, design, or flow (*n* = 467, 66.15%), both positively (*n* = 183, 25.92%) and negatively (*n* = 284, 40.23%), was the most frequently mentioned theme in the feedback. The largest dimension of the negative quality of assessment theme was poorly worded questions (i.e., were too long, overly complicated, lacked clarity; *n* = 150, 52.82%; *“Some of the questions would be a little vague and confusing at times, it could be useful to add an example after the question to explain what is mean”)*. A secondary dimension of the negative quality of assessment theme was related to the users’ ability to enter an appropriate answer (*n* = 110, 38.78%). This dimension consisted of users reporting missing relevant or accurate answer options, or an inability to select multiple relevant answer options (*n* = 83, 29.23%). Additionally, other users commented on the inability to enter qualitative data (*n* = 27, 9.51%) either in order to provide nuance alongside the answer option they selected, or as a stand-alone method to answer questions. The largest dimension of the positive quality of assessment theme was well worded questions (i.e., easy to comprehend, not overly intrusive; *n* = 175, 95.63%; *“The questions were very straightforward to follow so it was very nice tbh;” “Questions were good, easy to understand and give straight forward answers.”*).

Another frequent theme identified in the feedback related to the wording, design and flow of questions was the quality of the assessment flow (*n* = 193, 27.34%; see and Table 3), both positive (*n* = 90, 12.74%) and negative (*n* = 103, 14.59%). The most frequently mentioned dimension of the negative quality of assessment flow theme was repetitive questions (*n* = 90, 87.38%; *“Seemed fine, a little repetitive and as a result it felt a little like you were trying to catch me out. Which to be fair is possibly to try to control for recall bias, but the user experience felt a little taxing.”*).

**TABLE 3:** Frequency of theme combinations from thematic analysis of feedback responses about the question design, wording and flow, with ten or more instances in the dataset (*n* = 706; see Supplementary material 2 for the remaining theme co-occurrences).

Theme combination	Example feedback comment	Frequency (*n*,%)
Negative quality of assessment AND Negative usability	*“I felt some of the statements that I was required to rate how much I agreed with could have been simplified. I found I agreed strongly with one part of the statement but not at all with another part, which made it very hard to rate my level of agreement overall”*	46 (6.52%)
Positive quality of assessment AND Positive quality of assessment flow	*“Questions were worded well and flowed fluidly; I can see no obvious improvements which could be made.”*	31 (4.39%)
Negative quality of assessment AND Negative assessment flow	*“Some questions were rather repetitive or confusing in their wording.”*	27 (3.82%)
Positive quality of assessment AND Positive usability	*“[*…*] it was easy to access and the questions were easy to understand.”*	25 (3.54%)
Positive quality of assessment flow AND Positive usability	*“Worked well for the structure and flow on my mobile phone. Easy log in. [*…*]”*	10 (1.42%)
Negative quality of assessment AND Negative functionality	*“I accidentally selected the incorrect age for one question and it automatically moved into the next question. It would have been helpful to be able to go back and change this.* *Some of the questionnaires were not realistic regarding causes of periods of poor metal health (i.e., Having to confirm a singular cause such as physical)”*	10 (1.42%)

Almost all feedback in the positive quality of assessment flow theme referred to a good assessment flow (i.e., easy to follow, logical question flow, well grouped into question sections; *n* = 89, 98.89%; *“I thought the questions flowed very well. I didn’t mind doing them at all.”*).

The feedback comments also made reference to positive (*n* = 79, 11.19%) and negative (*n* = 105, 14.87%) usability of the assessment. Feedback comments mentioning positive usability features mainly focused on the assessment being well-designed (i.e., visually appealing; *n* = 42, 53.16%; *“Design of the website/app was very user friendly and smooth. Graphics were easy to understand.”)* and ease of use (*n* = 32, 40.51%; *“Simple and easy to use”*). Most feedback in relation to negative usability focused on the questions being hard to answer (*n* = 83, 79.05%). In some cases this was linked to the questions being poorly worded (*n* = 27, 18%; *“I felt some of the statements that I was required to rate how much I agreed with could have been simplified. I found I agreed strongly with one part of the statement but not at all with another part, which made it very hard to rate my level of agreement overall”*), and in other cases it was linked to the lack of ability to select an appropriate answer option (*n* = 19, 22.89%; *“Sometimes it was difficult to choose between two answers as it was difficult to decide which was most applicable.”*). However, some feedback comments cited the reason for difficulty in answering questions being due to, in some part, it being difficult to remember specific depressive or mania episode information (i.e., number of episodes, duration of episodes; *n* = 23, 27.71%; “*I think it’s very difficult to retrospectively our exact lengths of time on things and days per year. Lots of the time I’m not certain about how long I have been feeling things or extreme feelings I have subsided slowly. I also found it a little difficult to determine whether less extreme episodes were still classes as episodes*”).

Finally, negative functionality was also identified as a theme within feedback relating to the wording, design, or flow of questions in the digital assessment (*n* = 44, 6.23%). The vast majority of this feedback commented on the lack of a back button within the digital assessment, or the users missing the ability to review and amend their answers before submitting them for analysis by the algorithm (*n* = 41, 93.18%). Of users who provided this feedback, 31.71% (*n* = 13) reported that they were concerned about providing inaccurate data within the assessment as they were unable to amend their answers after choosing one (*“I feel like there should be a back button in case you answer a question incorrectly. At least one of my questions was answered incorrectly and I couldn’t change the answer”*).

Several themes overlapped within the feedback on the wording, design, and flow of the questions (Table 3). The most frequently overlapping themes were negative perception of quality of assessment and usability (*n* = 46, 6.52%). The majority of this overlap was between users reporting questions were hard to answer and (1) questions being poorly worded (*n* = 33, 70.21%; *“Some questions were very broad so quite difficult to answer especially with regards to symptoms.”*); (2) reporting an inability to answer appropriately (*n* = 21, 44.68%; *“Sometimes none of the multiple choice questions described my experiences so it was difficult to answer. Maybe some written/spoken answers more specific to a person would be more helpful.”*); or, (3) concerns about reporting inaccurate data (*n* = 15, 31.91%; *“Sometimes I honestly didn’t know how long specific conditions had been going on for or when my first episodes started or how long or how many I had over the years so I had to guess. I think my guesses were probably not even close to correct. There should be more opportunity for ‘I don’t know know”’*).

Other commonly overlapping theme combinations were a positive quality of assessment and the positive quality of assessment flow (*n* = 31, 4.39%), and a negatively perceived quality of assessment and assessment flow (*n* = 27, 3.82%; *“It was easy to answer and the questions flowed nicely”*). 24.44% (*n* = 22) of feedback which mentioned repetitive questions, also stated that questions were poorly worded. Users provided feedback that repetitive questions assessing the same symptom multiple times were worded too similarly, which caused reports of confusion from users (*“Word similar questions more contrastingly. Sometimes I didn’t know the difference between questions.”*).

Positive perception of quality of assessment and usability were also often identified in combination (*n* = 25, 3.54%) with many users commenting on both well-written questions and a well-designed assessment in their feedback (*n* = 23, 92.00%; *“They were laid out clearly and easy to understand”*).

#### Feedback on the homepage design and email reminders

A total of 671 relevant responses were included in the analysis of feedback on homepage design and email reminders. 389 (36.77%) responses were identified as being not applicable to the aims of the current study. The average word count of the included feedback for this question was 11.81 (*SD* = 11.13).

**FIGURE 5:**
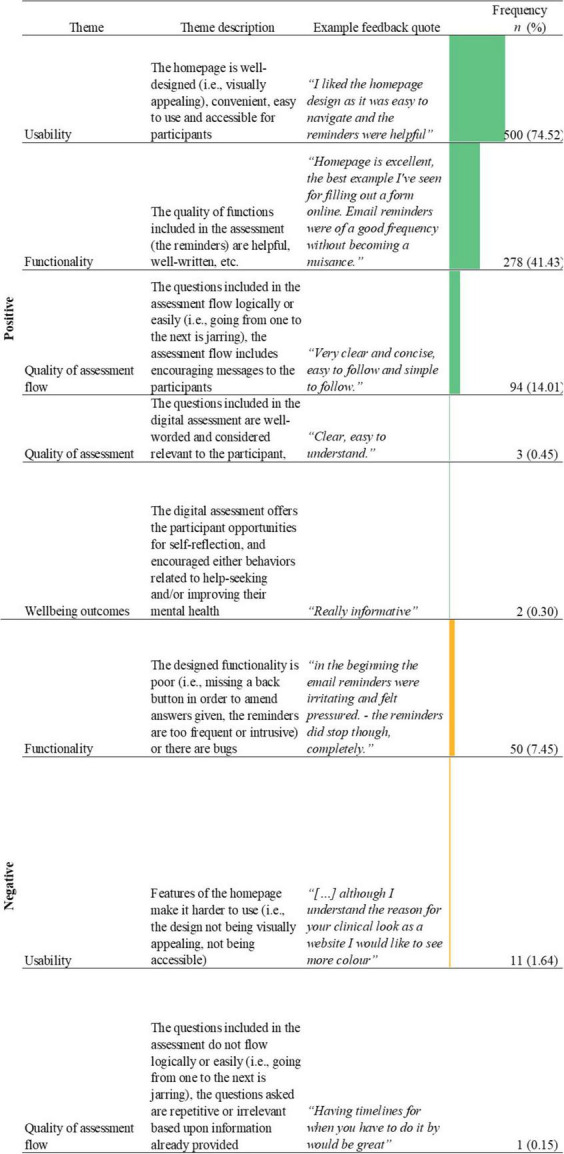
Name, description, examples, and frequencies of themes as identified in the thematic analysis of feedback responses about the homepage design and reminders (*n* = 671; See Supplementary material 1 for novel digital assessment theme count). Bars represent theme frequency as a percentage of the total number of relevant feedback responses (*n* = 671).

In total, eight themes were identified from feedback comments on the homepage and reminders included in the Delta assessment (See Figure 5), including two positive major themes and two negative major themes, focused upon usability and functionality.

When providing feedback on the homepage and reminders, the majority of feedback made reference to positive usability (Figure 5). The largest dimensions of this theme included ease of use (*n* = 307, 45.75%; *“Very helpful and easy to navigate”*) followed by a well-designed homepage (i.e., visually appealing, not overly cluttered, simple; *n* = 298, 44.41%; *“I really like these, the design is simple and attractive”*). There was overlap between the largest dimensions of the positive usability theme, with 122 (24.40%) feedback responses stating that the homepage was both well-designed and easy to use (*“Very user-friendly, uncluttered and straightforward. A*+”). Similarly, the negative usability theme (*n* = 11, 1.64%) was most frequently characterized by a poor design (*n* = 8, 72.73%; *“Good needs to be a little more mobile friendly”*).

In terms of functionality, the majority of feedback focused positively upon the reminders to continue the assessment (*n* = 278, 41.43%). Many users commented that they found the reminders to be helpful as a prompt to encourage them to continue with the assessment if they forgot to complete it (*“The homepage is really easy to navigate and the emails are helpful! Part of my mental health problems do concern forgetting things so they helped.”*). In contrast, of those who commented negatively on the email reminders (*n* = 33, 66.00%), users stated that the frequency of the reminders were irritating and made the user feel as if they were being pressured into continuing with the assessment (*“Toom many;” “[*…*] The email reminders are quite frequent which could put some users off, especially if they are in a depressive mood.”*). Other reported aspects of negative functionality included the presence of bugs (*n* = 15, 30%; *“The web app looked good. But there are things like being pieces to use portrait mode that stopped me doing things and had to keep putting it in desktop mode on my phone.”*) and a missing back button (*n* = 5, 10%; *“The one thing I found annoying was not being able to take a step backward if I made a mistake”*).

Several theme overlaps were identified in feedback related to homepage and reminders (Table 4). The most frequently identified theme overlap was between positive functionality and positive usability (*n* = 155, 23.10%). A large proportion of feedback which mentioned positive reminders, also mentioned that the assessment had a well-designed homepage (*n* = 121, 78.06%) and that the homepage was easy to use (*n* = 95, 61.29%).

**TABLE 4:** Frequency of theme combinations from thematic analysis of feedback responses about the homepage and reminders, with ten or more instances in the dataset (*n* = 671; see Supplementary material 3 for the remaining theme co-occurrences).

Theme combination	Example feedback comment	Frequency (*n*,%)
Positive functionality AND Positive usability	*“I liked the homepage design as it was easy to navigate and the reminders were helpful”*	155 (23.10)
Positive assessment flow AND Positive usability	*“Very easy to follow and get on with”*	36 (5.37)
Positive functionality AND Positive usability AND Negative functionality	*“Very user friendly and easy to use. Emails were helpful, sometimes too often”*	10 (1.49)
Positive functionality AND Positive assessment flow AND Positive usability	*“The website and emails were very well designed; everything was clear and understandable. The questionnaires all worked with no hitches.”*	10 (1.49)
Positive usability AND Negative functionality	*“worked well - nice that you can use it on a phone.* *an auto forward to the next question would be good, as well as the ability to go back to the previous question incase of a mistake”*	10 (1.49)

#### Feedback on the results report

179 (19.10%) responses to the question regarding the results report were identified as being not applicable to the aims of the current study. This left a total of 794 relevant responses. Of the relevant responses, the mean word count of the feedback was 27.85 (*SD* = 31.40).

**FIGURE 6:**
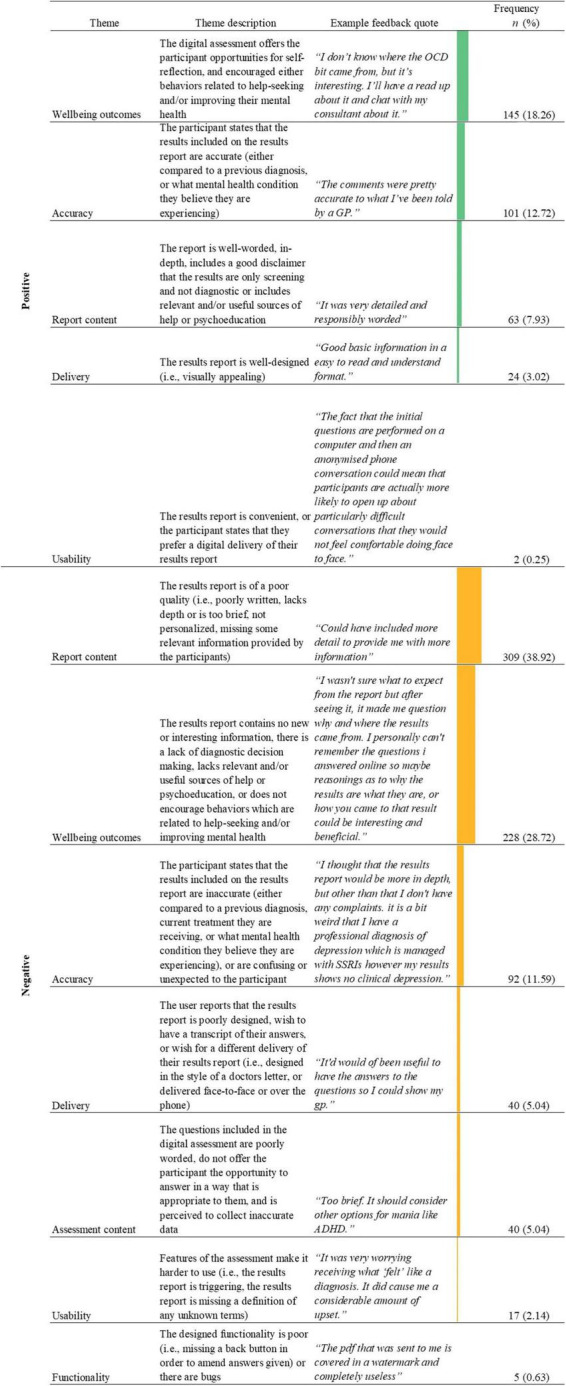
Name, description, examples, and frequencies of themes as identified in the thematic analysis of feedback responses about the results report (*n* = 794; See Supplementary material 1 for novel digital assessment theme count). Bars represent theme frequency as a percentage of the total number of relevant feedback responses (*n* = 794).

Through the thematic analysis, 12 themes were identified with three major positive and negative themes (see Figure 6).

The most common theme identified within the negative feedback commented on poor report content (*n* = 309, 38.92%), with feedback that the results report lacked depth comprising a major dimension of this theme (*n* = 279, 90.29%). Users reported that the content of the report was too brief (*“I would have liked a little bit more detail.”*). An additional common feature of feedback regarding poor report content was a lack of personalization of the report (*n* = 35, 11.33%; *“Not very useful or personal.”*). There was an overlap between feedback commenting the results report lacks depth and lacking personalization (*n* = 22, 7.12%; *“It was helpful to gain a diagnosis however I thought the report would be more comprehensive and personalized due to the level of information I provided [*…*]”*).

In contrast, 63 (7.93%) users provided positive feedback regarding the results report’s content. The majority of this feedback was centered upon the results report being of an adequate depth, and/or being well-worded (*n* = 54, 85.71%; *“It was very detailed and responsibly worded”)*. Users seem to positively comment on results report content when it was concise, detailed, or easy to understand. Of the users who stated that the results report was of adequate depth, and/or was well-worded, many commented that the results report was clear or easy to understand (*n* = 26, 48.15%; *“I found the results report easy to digest and understand. Thank you”*).

The second most commonly identified negative theme in feedback about the results report was negative wellbeing outcomes (*n* = 228, 28.72%). The major tenet associated with negative wellbeing outcomes was related to a lack of diagnostic decision making provided within the report, with many users stated that they wanted more detail on how the results report was reached (*n* = 128, 56.14%; *“Maybe should be clearer that it’s not that much information in it though that could be personal to me). I thought there might be more of a breakdown of why and how the conclusion was made [*…*]”*). A secondary aspect of the negative wellbeing outcomes theme was the user not considering the results report to be useful or to contain any new information about their mental health (*n* = 82, 35.96%; *“It didn’t tell me anything new or feel very detailed.”*). In some cases, the lack of usefulness or lack of new information was due to a lack of depth in the results report (*n* = 37, 45.19%; *“It was not detailed. I felt I had to answer a lot of personal questions just to receive a very generic response. It didn’t tell me anything i didn’t already know.”*). Additionally, within the negative wellbeing outcomes theme were comments stating that the results report was missing SOH or psychoeducation, or that the quality of these resources was low (*n* = 32, 14.04%; *“[*…*] information about the conditions that the questionnaire may have confirmed would have been welcome and more contacts for support, help and/or guidance would have been superb.”*).

Conversely, other users reported positive wellbeing outcomes (*n* = 145, 18.26%), the biggest dimension of which was the results report encouraging help-seeking or more health-conscious behaviors in users (*n* = 57, 39.31%). Most users commented on the results report’s ability to encourage help-seeking with users stating they planned to discuss their results with a clinician (*“Enlightening. I will be taking a trip to a GP.”*). Other users additionally stated that they thought the results report would help them initiate conversations with the clinician (*“It contained information that I had suspected for a little while. I may have more confidence to talk with my GP in further detail about my feelings now and explore further diagnoses.”*). In addition to saying that the results report encouraged help-seeking, a subset of these users (*n* = 5, 35.17%) stated that the results report was interesting to them or was useful in undisclosed ways (*“It was very simple, but it was useful to me to know that I don’t seem to meet the criteria for bipolar.”*

Users who mentioned positive wellbeing outcomes also provided feedback on SOH or psychoeducation (*n* = 29, 20.00%), stating that they considered it to be helpful and relevant (*“It was helpful to have help sources identified”*). Of people who noted that the results report contained high-quality SOH or psychoeducation, an equal proportion (*n* = 7, 24.14%) stated that the results report was well-worded (*“The results were presented well and easy to understand, I thought the additional support groups were a great idea.”*) or poorly worded (*“I feel like the results report wasn’t as comprehensive as I thought it would be. I thought it would be a bit more in depth. However, I think the suggestions on where to seek help and support is very good.”*).

Perceived accuracy was a major theme identified in feedback related to the results report, with users comparing the mental health conditions listed on their results report against either a previous formal (*“Is the same results as a psychiatrist had come up with, in my case it was very accurate.”*) or a self-suspected diagnosis (*“The results report helps me confirm the symptoms I suspect myself to have had.”*). More feedback was identified as commenting on the perceived accuracy of the results report (*n* = 101, 12.72%; *“Results report reiterated my diagnoses from my psychiatrist”*) than perceived inaccuracies of the results report (*n* = 92, 11.59%). The theme of negative accuracy was broader than that of positive accuracy however, encompassing both inaccuracy in the report (*n* = 62, 67.39%) and unexpected or confusing results on the report (*n* = 36, 39.13%), with these dimensions of negative accuracy theme overlapping (*n* = 14, 38.89%; *“Confused as I have been diagnosed bipolar but the results said I’m not”*).

The most frequent theme combination identified in relation to the results report were the negative report content and negative wellbeing outcome themes (Table 5). This theme overlap was frequently identified when users mentioned that the results report was both too brief and was missing information related to the algorithm’s diagnostic decision making (i.e., how a specific condition outcome was reached; *n* = 42; 64.62%; *“I found it too simple. I was hoping for more detail as to why these conclusions had been drawn.”*). Additionally, some feedback stated that due to the brief content of the results report it did not provide any new information beyond what users already knew about their mental health (*n* = 37, 56.92%;*“The results report was a bit brief; it didn’t really tell me anything I didn’t already know/suspect.”*). Finally, some feedback stated that the brief results report precluded users from help-seeking by not having enough detail in order to present it to a clinician (*n* = 5, 7.69%;*“It wasn’t at all as detailed as I thought it would be. I thought there would be an analysis of my answers not just ‘you might have depression’ and ‘you might have a panic disorder.’ I was hoping to take my report to my GP to help with getting and accurate diagnosis for my poor mental health but I feel the report will be useless.”*).

**TABLE 5:** Frequency of theme combinations from thematic analysis of feedback responses about the results report, with ten or more instances in the dataset (*n* = 794; see Supplementary material 4 for the remaining theme co-occurrences).

Theme combination	Example feedback comment	Frequency (*n*,%)
Negative report content AND Negative wellbeing outcomes	*“I think it would be more interesting and helpful to have a more comprehensive report on the results. I agree it wouldn’t be right to give a diagnosis this way but more information on how you came to your conclusions and more in depth knowledge for my GP would be helpful.”*	65 (8.19)
Positive accuracy AND Positive wellbeing outcomes	*“With my results I can now go to my doctors and explain what i did as the report seems to be correct as it has highlighted something that we mentioned before to the doctors”*	18 (2.27)
Negative assessment content AND Negative accuracy	*“As I said about the questions this meant you don’t get a true picture of me thus you can’t give a correct analysis of me.”*	18 (2.27)
Negative wellbeing outcomes AND Positive accuracy	*“Results report wasn’t particularly helpful to me as I’m already being treated for depression. However it did confirm what I already knew”*	10 (1.26)

The themes of positive accuracy and positive wellbeing outcomes were also commonly identified in combination (*n* = 18, 2.27%). Much of the feedback which reported both positive accuracy and positive wellbeing outcomes, indicated that the results report was useful or interesting (*n* = 10, 55.56%; *“Useful to know, confirms a suspicion I’ve had for a while.”)*. An additional proportion of feedback which mentioned positive accuracy and positive wellbeing outcomes in combination mentioned that the results report encouraged help seeking behaviors (i.e., encouraged users to take their results report to discuss with a clinician; *n* = 8, 44.45%).

The negative assessment content and negative accuracy themes were also identified frequently in combination (*n* = 18, 2.27%), with users stating that the results included in the report were inaccurate due to the assessment omitting relevant information (i.e., a more in-depth assessment of past psychiatric history, how well medication is managing their psychiatric symptoms, information about menstruation and its impact on the user’s mental health, other diagnosed mental health conditions the user has which may be misidentified as a different condition by the algorithms; *n* = 17; 94.45%; *“The report I was given tells me I am bipolar when in fact I am menopausal which give similar symptoms at times.”*).

Some feedback commenting on the positive accuracy of the results report also commented on negative wellbeing outcomes, with users reporting that the results report was useless or lacked any new information as it confirmed a previous diagnosis (*n* = 18; 21.95%; *“I would’ve liked more detail in my results. I already knew I had bipolar and anxiety, I like that there was information attached but it was very generic. It would’ve been nice to see if I had particular tendencies that would respond to certain types of help more than others.”*).

#### Feedback on the composite international diagnostic interview

A total of 84 feedback responses mentioned the CIDI unprompted, across all three of the feedback question prompts. 81 (96.43%) of the CIDI feedback was in response to the prompt about the design, wording, or flow of the questions mentioned the CIDI.

**FIGURE 7:**
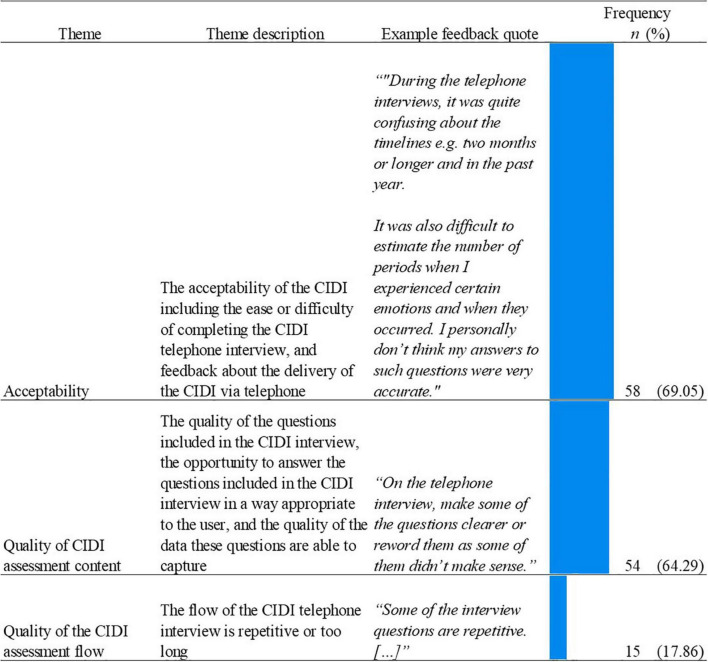
Name, description, examples, and frequencies of themes as identified in the thematic analysis of unprompted feedback responses about the CIDI (*n* = 84; See Supplementary material 5 for CIDI theme count). Bars represent theme frequency as a percentage of the total number of relevant feedback responses (*n* = 84).

Three themes were identified from the thematic analysis of feedback comments which mentioned the CIDI telephone interview (Figure 7).

The most frequently identified theme related to the CIDI was the acceptability of the CIDI telephone interview (*n* = 58, 69.05%). The most frequently mentioned dimension of this theme was the CIDI containing hard to answer questions (*n* = 39; 67.24%; *“[*…*] It is also quite difficult to recall in the moment specific (or first) episodes and exactly how long they lasted.”)*. A secondary dimension was related to the delivery of the CIDI via a telephone call, with users stating they would have preferred a different delivery (*n* = 14, 24.14%; *“The phone interview was extremely long and could have been done via online. [*…*]”*). Of those users who stated they would have preferred a different delivery, some commented a preference for completing the CIDI on an online platform (*“The telephone survey could have been done online. It would have made it easier for me to think back to historic episodes.[*…*]”*) or requesting the ability to view a copy of the questions before or during the phone interview (*“The wording of the questions was okay, but it may have been beneficial as a participant to have a copy of the interview questions and answers.”*).

The acceptability theme was closely followed by the theme of the quality of CIDI assessment flow (*n* = 54, 64.29%). The majority of feedback within this theme was negative, with a focus on poorly worded questions (*n* = 32, 59.26%; *“The questions in the phone call are far too long to keep a high level of focus”*) and users reporting concerns that the CIDI was collecting inaccurate information (*n* = 17, 31.48%; *“A lot of the clinical telephone questions were very hard to answer accurately, e.g., asking for exact numbers of depressive and high episodes, for exact lengths of each episode, to rate exact numbers for hours of sleep etc., and a lot of the questions were difficult to give just a yes or no answer to”*).

Finally, of the users who provided feedback on the CIDI, 15 (17.68%) commented on the quality of the interview flow. Similarly to the digital assessment, many of the users who provided feedback on the flow stated that the questions asked were repetitive (*n* = 10, 66.675; *“Phone interview quite repetitive”*). Additionally, users also commented that the CIDI interview was too long (*n* = 6, 40.00%; *“Clinical interview is too long.”*).

Only two themes were frequently identified in combination within unprompted feedback regarding the CIDI (Table 6). The themes of quality of assessment and acceptability were identified more frequently in combination (*n* = 32, 38.10%) than in isolation (*n* = 19, 22.62%; and *n* = 18, 21.43% respectively). Within this theme overlap, the majority of feedback stated that the questions were both poorly worded and hard to answer (*n* = 10, 52.63%;*“Some questions were too broad up to interpretation and were hard to answer as it wasn’t clear what the exact question was and the phone interviewer is unable to explain further”*).

**TABLE 6:** Frequency of theme combinations from thematic analysis of unprompted feedback responses about the CIDI, with ten or more instances in the dataset (*n* = 84; see Supplementary material 6 for the remaining theme co-occurrences).

Theme combination	Example feedback comment	Frequency
CIDI quality of assessment content AND CIDI acceptability	*“They were quite wordy so would have been easier to do online rather than the phone, e.g., in how many separate years have you had had episodes that last for 4 days or longer in which you feel a, b, and c. Would have been easier to have in front of me to reread and be sure I understood and answered correctly.”*	32 (38.10)

## Discussion

### Overview

The primary aim of the current study was to utilize thematic analysis methods of user feedback in order to determine features of a novel digital health assessment which user perceived as either positive or negative. This aim was conceived with the view to offer recommendations in improving the user experience of digital mental health self-assessments. Feedback indicates that the majority of participants who completed the digital mental health assessment considered it to be worthwhile, with over a third categorizing it as very worthwhile. This finding corroborates previous evidence demonstrating that users find engaging with DMHI to be helpful (81, 82). It also supports research that found, via varied measures, high satisfaction is reported by individuals using mental health apps.

Despite the majority of participants considering the current digital mental health assessment to be worthwhile, the thematic analysis indicates key areas for improvement. Most of the written feedback commenting on both the design, wording and flow of the questions, and the results report was negative. This finding may be explained by the framing of the prompts delivered in the feedback survey. The questions were designed to elucidate actionable, constructive feedback on the digital mental health assessment. Therefore, the written feedback was expected to include feature suggestions to improve the assessment.

### Themes identified in feedback of a novel digital mental health assessment

Overall, across all feedback questions, the major themes identified in relation to the digital mental health assessment appeared to be the quality of the assessment, its usability, the quality of the report content, accuracy, and wellbeing outcomes. Additionally, functionality was identified as a minor theme across all of the feedback question prompts.

Within the feedback prompt for the question wording, flow, and design of the questions, two-thirds of the feedback was related to the perceived quality of the assessment. Within this theme, the majority of feedback was negative, indicating that questions included within the assessment were overly complex or too long. This appears to be a feature of both psychiatric assessments investigated in the current study, as this feedback was also identified in relation to the CIDI. This negative sentiment regarding the quality of the questions emerging from this feedback indicates the importance of engaging extensively and widely in Public and Patient Involvement (PPI) activities and co-design. PPI involves research being conducted “with” patients and/or the public in an active participatory relationship, rather than “for” patients and/or the public by researchers (83). PPI work can be widely varied including tasks such as defining research priorities (83), and reviewing the study design (84). Engaging in PPI activities within research is now considered best practice (85), with the combination of expertise through experience of a mental health disorder and expertise through clinical experience suggested to facilitate the best design and provision of mental healthcare services (86). As an example, a co-design of mental healthcare services between staff, patients and carers has been demonstrated to improve the quality of acute care services (87). Of interest to the current study focus, a systematic review mapping the impact of PPI on health and social care research demonstrated that PPI can lead to higher quality of research materials, including improved wording of research questionnaires (88). Including PPI panels in the development of questionnaires can enhance the validity of the questions asked and ensure a comprehensive question set (88). Any opportunity to improve the validity of questions asked in a digital mental health assessment should be pursued, particularly as a systematic review of the validity of digital psychiatric assessment tools is lacking high-quality evidence (24).

Whilst a patient panel was consulted in relation to all aspects of the development of the Delta Study assessment tool prior to the pilot study, and amendments were made to the novel digital assessment and study materials after receiving feedback, the feedback demonstrates there are still areas with scope for improvement. Therefore, by engaging in as much PPI tasks as possible, many different perspectives can be captured in the design stage in order to maximize the likelihood of designing an assessment which is both accessible and usable to all in the intended population. Ensuring the questions are easily comprehended by users who are experiencing mental health symptoms is particularly important when considering possible cognitive/concentration difficulties associated particularly with low mood, MDD (68, 89) and bipolar disorder (90). This is important as previous literature suggests that impaired neurocognitive functioning may be associated with poorer ability to engage in digital health tools for mental health conditions (91). However, it should be noted that this is not isolated to engagement with DMHIs, as in order for patients to engage with traditional care they must also be able to engage in a lengthy diagnostic interview (92). The advantage of DMHIs is that the assessment can be completed at an opportunity most convenient to the user, and when the user feels most concentrated, potentially encouraging better engagement.

Another dimension of the quality of the assessment was related to a reported lack of ability to select appropriate answers. This was reflected in some users stating that a necessary answer option was missing or that they were unable to choose multiple answer options when needed. Therefore, considering that often symptoms are hard to quantify, only offering pre-defined answer options may make it hard to fully capture the severity and psychological burden. Associated with an inability to select an appropriate answer were concerns that the data collected may be inaccurate as users felt they were encouraged to select the “closest fit” answer option, as the most accurate answer option was not available. Considering the core motivation for completing a digital mental health assessment is likely to be receiving an accurate indication of current mental health status, a perception that inaccurate data is being collected may be a barrier to engagement. Developers should aim to engage users thorough PPI activities to ensure that a large range of answer options are available to users to best allow them to reflect their experiences and symptoms. Additionally, developers may wish to consider offering multiple modalities of answer entry, chiefly open or free text boxes. In the current study, some users requested the ability to enter other datatypes such as free text in order to provide personal context. These free text boxes could be implemented alongside more standardized measures to maintain the collection of necessary data to assess symptom fit against diagnostic criteria. Aside from being a commonly requested feature identified in the current study, the addition of free text would allow for potentially richer data which could be used for additional diagnostic decision making such as during a clinician review of questionnaire data.

Usability is a commonly reported feature of user feedback reported in previous literature (58) and is a potential barrier to usage of digital tools (59). An attractive design and ease of use are the highest rated aspects to user engagement with DMHIs identified in user reviews (27, 92). Both of these dimensions of usability were identified within the current study in relation to feedback on the homepage design and reminders. Ease of use and good visual design were mentioned in the majority of feedback response within the usability theme, and were often identified in combination indicating that high-quality visual design will contribute to ease of use.

Additionally, in the current study we found that usability extends to the question wording, flow and design with some users reporting that the questions were hard to answer, however this is likely not exclusive to only mental health questionnaires. In some cases, the reporting of difficulties in answering questions was due to the questions assessing hard to qualify and quantify psychiatric symptoms, or difficulties in remembering episode details (i.e., frequency and duration of depressive and/or manic episodes, the severity of past symptoms). Within the context of bipolar disorder for example, previous literature demonstrates patients experience difficulties in recall of previous (hypo)manic episodes and symptoms (93, 94). This potential difficulty in providing answers to questions could impact both the engagement with the digital tool and the accuracy of any given results, with some users reporting having to make guesses if they did not have a good recollection of their symptomatology. This is a concern in all deliveries of psychiatric assessments, including in-person, as identification of mental health disorders relies upon the patient’s narrative and insight. Future work may consider investigating to what extent a patient’s level of insight impacts the accuracy of reporting symptoms, and in turn the assessment outcomes in order to determine to what extent this needs to be controlled for during digital (and indeed in-person) psychiatric assessments.

Functionality, whilst being a minor theme identified in the current study, was highly related to usability in terms of the homepage design and reminders. The major aspect of functionality commented upon in the current study was reminders, with more users positively commenting on the quality of the reminders. This is in line with previous findings, which also found that reminders are perceived as a positive feature of mental health apps (95), and are a commonly requested app feature by users (92). In fact, receiving reminders to interact with a DMHI is also associated with higher engagement (59). Additionally, the inclusion of reminders in apps for mental health may support users who are experiencing cognitive symptoms associated with mental health disorders, primarily poor memory and concentration (68, 89). On the other hand, some users reported that they perceived the reminders to be negative, stating that the reminders were sent too often and became intrusive. Therefore, developers may consider allowing the users to modify the number and frequency of reminders they receive to best meet their personal preferences. This is supported by previous evidence highlighting user preference for customizable reminders (57, 58). Considering that integration of DMHIs into user’s daily life is considered a facilitator to engagement (59), providing users with the ability to personalize aspects of the reminders (i.e., number of reminders, the time of day) may encourage engagement.

The quality of the report content was a major theme identified both in a positive and negative context in response to the report feedback prompt. The majority of negative feedback was related to the report lacking adequate depth, with users requesting more symptom details alongside the likely diagnosis indicated by the algorithm to reflect the amount of information they provided during the assessment. Additionally, some users indicated that the results report was lacking personalization. Previous literature demonstrates the importance of personalization from analyses of user reviews (58). Further reports posit that a lack of perceived personalization in DMHIs constitute a barrier to user engagement (59, 96), and that offering personalized feedback increases user engagement (97). Therefore, by ensuring that the report content is personalized to the data provided by the user during the completion of the assessment, engagement may be increased. The constructs of the results report lacking adequate depth and lack of personalization were also identified in combination, with users reporting expectations of the report being an in-depth analysis and personalized to the responses they provided. The importance of personalization is further reflected in the current study, as some users commented that the provided SOH and psychoeducation were a positive feature when perceived to be relevant to their results, and likewise considered a negative wellbeing feature if relevant SOH or psychoeducation was missing.

Conversely, positive report content was centered on the report being well-worded and of an adequate depth. Whilst this was a smaller proportion of provided feedback, it is still of interest that different users can hold different sentiment polarity opinions toward the same report content. Therefore, future work should consider investigating factors related to the level of detail in a mental health results report a user prefers. This would also offer the opportunity to expand the ability for personalization to user preferences, by offering the user their preferred level of report detail. Investigating user-centered factors related to determining the user’s preferred level of results report detail is further substantiated by the overlapping themes of poor report content and negative wellbeing outcomes. The current study observed that a perceived lack of detail in the results report was associated with a lack of new or useful information being provided to the user. Future work may wish to investigate whether users with prior knowledge of their own mental health or previous interactions with mental health services may require additional detail as compared to users who do not have such past knowledge or experience. This would assist in ensuring all users are offered meaningful and actionable insights from the results report, irrespective of their prior level of knowledge of their own mental health. In the current study, accuracy was identified as a theme in relation to the results report, with a similar proportion of users perceiving the results as accurate or inaccurate when comparing them to either self-suspected or previous formal mental health diagnoses. Importantly, when assessing accuracy of mental health assessments some users seem to use their own self-suspected diagnosis as a “gold standard.” More people now look for information about mental health online and evidence indicates that among individuals who search for a potential diagnosis online, thirty-five percent did not visit a clinician to confirm their diagnosis (98). This suggests that some users potentially utilize a digital psychiatric assessment for confirmation of a self-suspected diagnosis, rather than seeking an assessment from a clinician. The potential dangers associated with self-diagnosis of mental health conditions are a great concern (99), especially if coupled with potential self-medication through illegal online drug providers. Therefore, future work should consider exploring the incidence of users who utilize a self-suspected mental health diagnosis as a gold standard when assessing the accuracy of a digital mental health assessment, or online symptom checker.

The current study demonstrated that completing a digital mental health assessment is associated with both positive and negative wellbeing outcomes, as perceived by users. In terms of positive wellbeing outcomes, the major dimension was related to the receipt of a results report encouraging help-seeking, or facilitating more health conscious behavior to improve their mental health. This finding reflects insights from previous reports demonstrating that engaging in online screening tools increases one’s likelihood in seeking mental healthcare or support (82, 100–102). Additionally, some users stated that they considered their results report to be of interest or of non-specified usefulness. This reflects previous work which demonstrates that an increase in mental health understanding and/or responsibility is a commonly identified feature mentioned in app store reviews of mental health apps which offer an assessment. Therefore, whilst some users did not indicate that they took their results report to a clinician to discuss, this aspect of the positive wellbeing outcomes theme suggests that the results report can still offer value outside of a formal healthcare context.

The core dimensions of the negative wellbeing outcomes theme was related to a lack of explanation of diagnostic decision making within the results report. This has also been identified in previous reports in relation to Artificial Intelligence (AI), which highlighted that users of online symptom checkers wish to be provided an explanation for the results reached based upon their personal data (103). Ensuring that users are aware of how results of digital assessments were reached may potentially increase trust, and encourage users to follow personalized triage recommendations (104). This was also reflected in the findings of the current study, which showed that some users reported that the lack of explanation of diagnostic decision making precluded them from or caused hesitation in showing their results report to a clinician.

### Themes identified in the composite international diagnostic interview feedback

The most frequent theme identified in feedback on the telephone-delivered CIDI was acceptability. Similar to the feedback for the digital assessment, the current study identified that poor acceptability of the CIDI was also related to the included questions being considered hard to answer by users. Therefore, this further demonstrates that difficult to answer questions are not simply a feature of digital mental health screening tools, and also extend to interviewer-led telephone interviews.

Interestingly, in relation to the theme of acceptability some users stated they would have preferred a digital rather than phone delivery of the CIDI, while the majority did not comment on it. This reflects previous work demonstrating that when a computerized version of the CIDI is delivered, 94 percent of 222 patients in an acute psychiatric setting liked the interview, with a further 60 percent stating that they found the interview just as comfortable or more comfortable than completing an in-person interview with a doctor (105). However, these findings must be evaluated with caution since this previous work was published over 20 years ago and the digital literacy of the population has increased. Similarly, our study was not focused on the CIDI and the feedback gathered in relation to it was incidental; hence, further work is required to investigate the suitability of the CIDI for digital delivery.

The second most frequently identified theme related to the CIDI was the quality of the assessment content. Some users who underwent the CIDI assessment stated that the questions included in the assessment were poorly worded or lacked an appropriate option to provide an accurate answer. Similar to the current study, previous work also identified a lack of suitable answer options as a frequent criticism of the CIDI (105). However, this may be a feature related to the previously stated difficulties of quantifying and qualifying highly heterogenous and subjective mental health symptoms into the “neat” binary categories often employed for data collection with screening tools or in a structured interview. This again highlights the potential benefits conferred by offering users free text modalities to provide additional information as well as using pre-defined questions and answer options to determine fit to diagnostic criteria.

### Limitations

Despite the learning offered from the current study, the content and sentiment of the feedback of both the novel digital mental health assessment and CIDI may have been skewed due to several reasons.

Firstly, unfortunately, as the feedback questions were only asked to users who completed the entire digital assessment, and received their results report, no feedback was collected from users who dropped out. Therefore, the feedback sentiment or content may be skewed, and does not capture the reasons of users who have dropped out. However, as we included a large dataset of users who completed the entire digital assessment we have an evidence base demonstrating features which users who completely engaged with a digital mental health assessment considered to be positive or negative. Despite this, future work may consider addressing this by providing all users, even those who drop out, the opportunity to offer feedback. The study cohort only included individuals experiencing symptoms of low mood, or with a previous diagnosis of a mood disorder (i.e., MDD or bipolar disorder). Thus, the findings may not be representative to other patient populations outside the scope of the original pilot study. Therefore, caution should be taken when applying the findings to non-psychiatric users or users with other mental health concerns or disorders. Future work should address these populations, by assessing feedback on the same domains as the current study.

Within the current study, there were no group differences in terms of gender between Delta Study participants who did and did not provide feedback; however, the results of the current analysis of this written feedback should be interpreted with the caveat that the majority of participants were female. There is substantial evidence that, despite disproportionately high rates of suicide in men compared to women (106), men are less likely to seek help or engage in psychiatric treatment either in-person or through DMHIs. This is corroborated by evidence of low rates of help-seeking for mental health concerns in men (107). Some explanations for these low rates of help-seeking include stigma, adversity to appearing “vulnerable,” and difficulties in effectively communicating mental health concerns with healthcare professionals (108). Several of the above mentioned barriers to help seeking can be addressed with DMHIs, however the majority of users of such tools appear to be women (59). Future work should investigate which features and delivery methods would encourage men to engage with DMHI’s and in turn with mental health support and treatment. The thematic analysis method employed in the current study is potentially susceptible to bias. However, by implementing an independent double review for each piece of feedback and for each identified theme, we aimed to mitigate this risk.

Finally, there was no specific question assessing user perspectives of the CIDI and its mention was incidental. Therefore, the views expressed may not represent the full spectrum of perspectives of individuals who completed the CIDI. This may explain why the feedback given in reference to the telephone-delivered CIDI was overall negative. Future work may wish to investigate user experiences of the CIDI, and opinions toward different modalities of its delivery in order to determine how best to utilize the tool within both clinical and research settings.

### Recommendations for improving user engagement

1. Engage in extensive and iterative PPI activities, ensuring a wide range of patient perspectives are captured to establish that questions and associated answer options included in mental health assessments are of an acceptable quality and quantity to both (1) improve user experience, (2) certify the validity and comprehensiveness of the assessment, and (3) enable users who are experiencing cognitive symptoms associated with mental health disorders to be afforded equal participation opportunities.
2. Consider offering multiple modalities to answer questions within the assessment, such as free text boxes with a view to both (1) increase user engagement; and (2) use this additional data to further inform diagnostic decision making, such as via clinician review.
3. Include reminders to encourage the user to complete their assessment, whilst providing the opportunity for the user to personalize reminder frequency.
4. Consider providing information on the algorithm’s diagnostic decision-making logic, to both (1) increase users’ trust in the results and (2) increase the likelihood of users sharing their results with a healthcare provider.
5. Ensure that the results provided following a digital mental health assessment are in-depth enough to be actionable by users (i.e., in-depth enough for the user to feel comfortable to share with their healthcare provider), and reflect the amount of time the user has invested to complete their assessment.
6. Provide relevant information, SOH, and psychoeducation which is personalized to the results of the digital mental health assessment.
7. Future work should consider investigating the acceptability of digitally delivered structured diagnostic interviews, such as the CIDI, in light of the current landscape of a more digitally native population.

## Data availability statement

The original contributions presented in the study are included in the article/Supplementary material, further inquiries can be directed to the corresponding author/s.

## Ethics statement

The studies involving human participants were reviewed and approved by the University of Cambridge Human Biology Research Ethics Committee (approval number HBREC 2017.11). The patients/participants provided their written informed consent to participate in this study.

## Author contributions

SB conceived the Delta Study, which provided the data for the current study. EF conceived the focus of the current study. EF was performed the qualitative analysis as first reviewer and BS and JB as the second reviewers. EF prepared the manuscript with revisions from BS, JB, NM-K, TM, GB-O, TO, and SB. All authors contributed to the article and approved the submitted version.
